# Gallbladder malignancy in the setting of Mirizzi syndrome with bile duct involvement: a case report

**DOI:** 10.1093/jscr/rjag723

**Published:** 2026-08-21

**Authors:** Anthony A Poluyanoff, Ebunoluwa A Ajadi, Dylan Flood, Arnold D Salzberg

**Affiliations:** Virginia Tech Carilion School of Medicine, 2 Riverside Circle, Roanoke, VA 24016, United States; Department of Surgery, Roanoke Memorial Hospital, Roanoke, VA 24016, United States; Department of Surgery, Roanoke Memorial Hospital, Roanoke, VA 24016, United States; Department of Surgery, Roanoke Memorial Hospital, Roanoke, VA 24016, United States

**Keywords:** gallbladder cancer, Mirizzi syndrome, cholecystitis, hepaticojejunostomy, ERCP

## Abstract

Mirizzi syndrome is a rare complication of gallstone disease characterized by extrinsic compression of the common hepatic duct and is associated with an increased risk of gallbladder carcinoma. We report the case of an 83-year-old female who presented with altered mental status, cholestatic hepatic enzyme elevation, and obstructive jaundice. Computed tomography imaging demonstrated biliary ductal dilation with a proximal common bile duct stricture concerning for malignancy. Intraoperative findings during laparoscopic cholecystectomy confirmed Type I Mirizzi syndrome with suppurative cholecystitis. Histopathology revealed well-differentiated adenocarcinoma involving the gallbladder neck with positive margins. The patient subsequently underwent definitive resection with completion cholecystectomy, central hepatectomy, bile duct resection, periportal and perihepatic lymphadenectomy, and Roux-en-Y hepaticojejunostomy. This case highlights the diagnostic and operative challenges posed by Mirizzi syndrome-associated biliary malignancy and underscores the importance of multidisciplinary management in achieving successful oncologic resection.

## Introduction

Gallbladder cancer is the most common biliary tract malignancy but remains rare in the general population, making up 12 640 of the 2.1 million new cancer cases diagnosed in the USA in 2026 and causing 4590 deaths [1]. Over two-thirds of gallbladder cancers occur in women, and incidence increases with age [2]. Many patients present with advanced disease, and ~50% of cases are discovered incidentally during cholecystectomy [3]. Most gallbladder cancers are adenocarcinomas, and the vast majority arise spontaneously [4]. In rare cases, chronic severe inflammation from underlying biliary tract obstruction predisposes patients to developing gallbladder malignancy. Mirizzi syndrome is a rare complication of gallstone disease characterized by extrinsic compression of the common hepatic duct by a stone obstructing the cystic or Hartmann duct [5]. Occurring in only 0.1%–2.8% of patients with gallstone disease in several large retrospective cohort studies [6–8], patients with Mirizzi syndrome have a significantly elevated incidence of gallbladder carcinoma compared to those with uncomplicated gallstone disease [9–11]. We describe the case of an 83-year-old female who presented with obstructive jaundice and was subsequently found to have Type I Mirizzi Syndrome during cholecystectomy, along with a stricture at transition between the common bile duct (CBD) and common hepatic duct (CHD). Sampling of the CBD and gallbladder revealed well-differentiated adenocarcinoma in the gallbladder neck. She subsequently underwent laparotomy to remove remnant infundibulum and cystic duct, along with partial hepatectomy and periportal/perihepatic lymphadenectomy with Roux-en-Y hepaticojejunostomy (HJ).

## Case report

**Figure 1:**
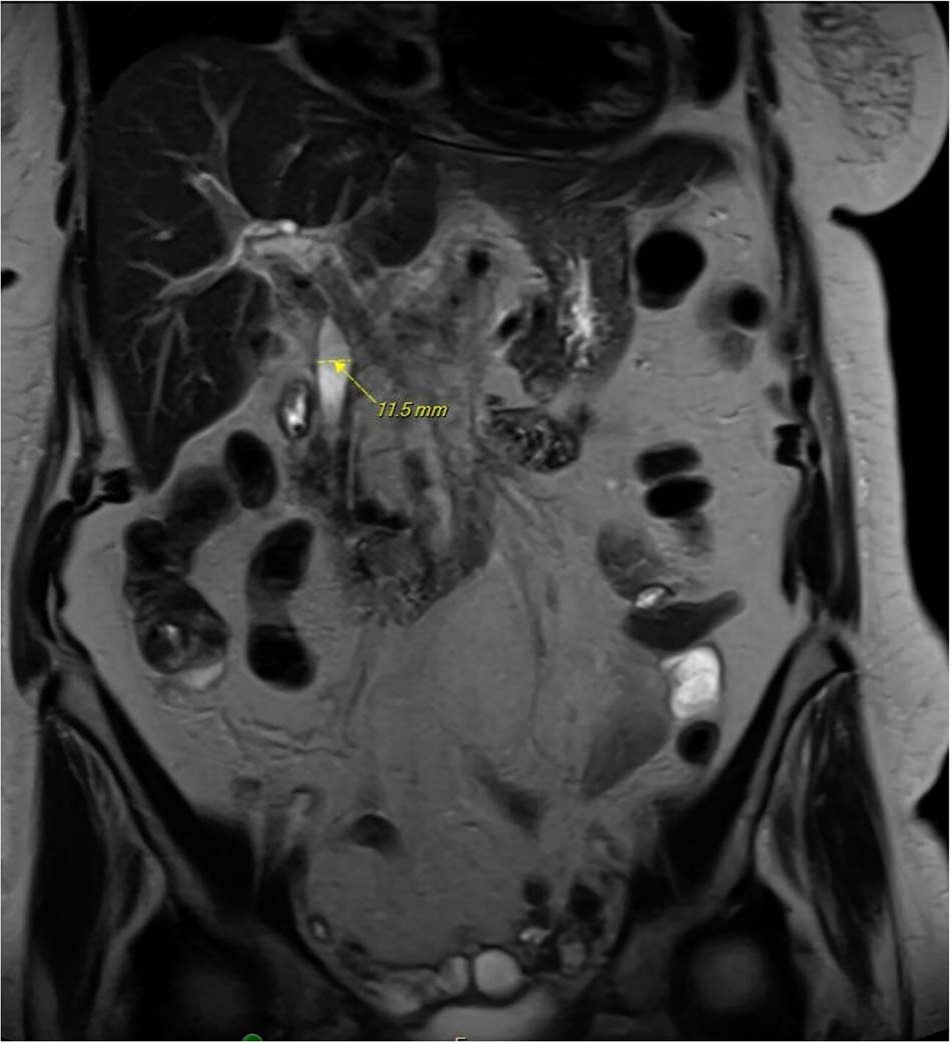
Coronal MRI showing a dilated CBD, highlighted by the arrow, without a discrete mass.

**Figure 2:**
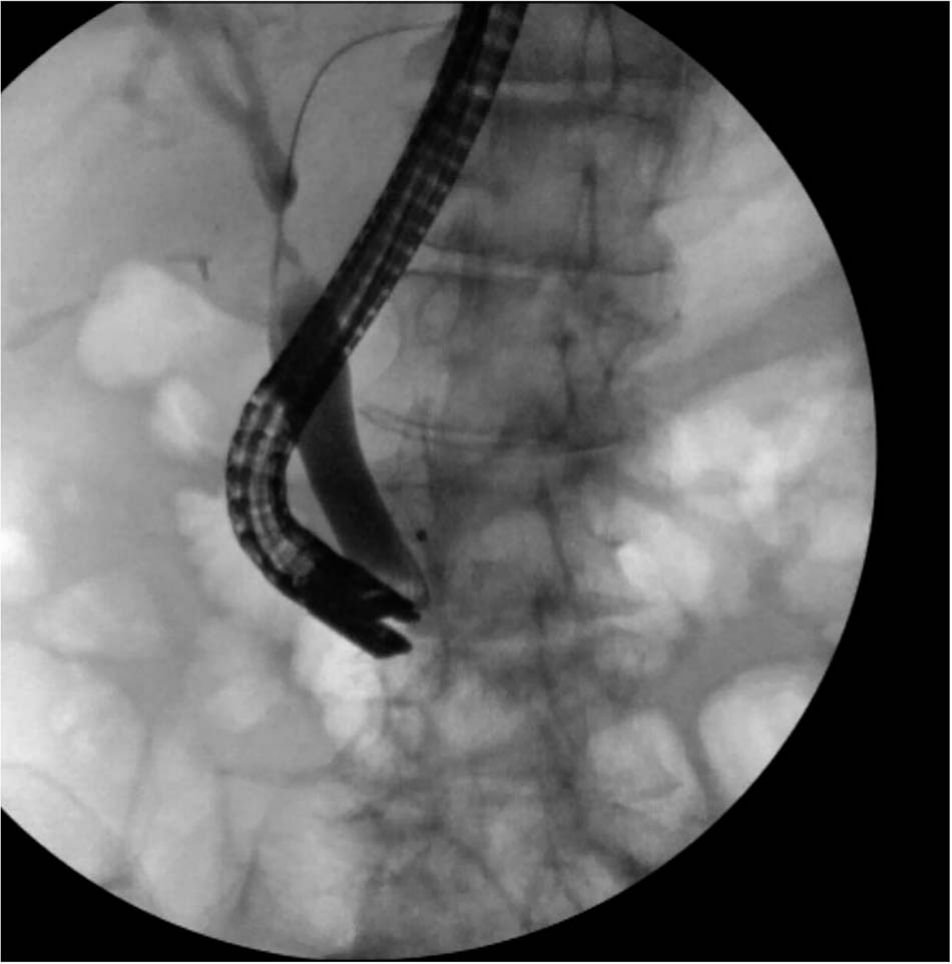
IOC obtained via ERCP highlighting a stricture at the level of the CHD; this image was taken during the patient’s initial cholecystectomy.

**Figure 3:**
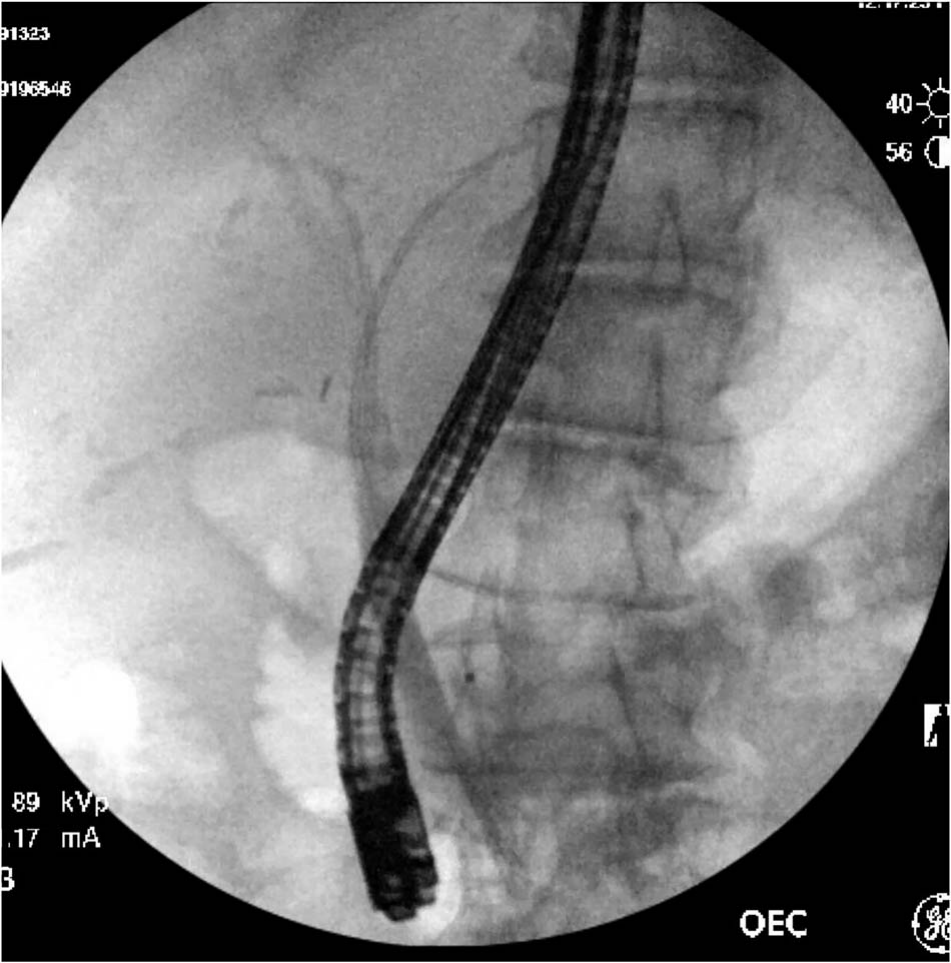
IOC via ERCP showing stents in the CBD and secondary stents in the hepatic ducts.

An 83-year-old female with no prior abdominal surgical history presented to an outside hospital (OSH) with a chief complaint of altered mental status. Symptoms had rapidly worsened over the previous 2 weeks, and she reported nausea, vomiting, and chills shortly before presenting to the OSH, with an episode of epigastric pain the previous night. Labs showed a white blood cell count of 11.0 K/ul and significant transaminitis, with aspartate transaminase, alanine transaminase, and alkaline phosphatase of 326, 275, and 626 U/l, respectively, along with a total bilirubin of 3.9 mg/dl. Computed tomography (CT) imaging of the abdomen revealed a distended gallbladder with intra and extrahepatic biliary ductal dilation with focal narrowing and wall thickening of the proximal CBD, interpreted as stricture vs. cholangiocarcinoma. CT imaging of the head was negative for any acute process. Magnetic resonance imaging (MRI) performed post-transfer at our center confirmed a distended gallbladder with stones, along with wall thickening and fluid, and confirmed a CBD stricture without an identifiable mass (Fig. 1). She subsequently underwent a laparoscopic cholecystectomy with intraoperative cholangiogram (IOC). Operative findings were suppurative cholecystitis with a large stone in the cystic duct, along with a stricture at the transition point of the CBD to CHD, confirming Type I Mirizzi syndrome. Endoscopic retrograde cholangiopancreatography (ERCP) was performed intraoperatively along with pancreatic sphincterotomy, which demonstrated the stricture that was subsequently stented (Fig. 2). Pathology of the gallbladder revealed subserosa-invading well-differentiated adenocarcinoma in the setting of high-grade dysplasia (HGD) with positive cauterized surgical margins and extension of HGD into Rokitansky–Aschoff sinuses. The biliary primary tumor site was indeterminate due to overlapping morphological features of the submitted sample. This sample was identified as including the gallbladder neck without a visualized cystic duct, and final grading was pT2a with probable liver parenchymal involvement. The patient recovered well and was evaluated for oncological resection 1 month later. She underwent an intraoperative ERCP with choledochoscopy for stent removal and biopsy of the CHD, after which she underwent an exploratory laparotomy with a segment 4B and 5 central hepatectomy, perihepatic and periportal lymphadenectomy, resection of the CHD and CBD, HJ, completion cholecystectomy, and hepatic duct stent placement. Intraoperatively, a dense stricture was found at the middle of the CBD distal to the entry of the cystic duct, and stents were placed in the CBD and hepatic ducts to relieve biliary obstruction prior to definitive management (Fig. 3). Severe inflammation from the prior partial cholecystectomy complicated definitive resection. The CBD and cystic duct were resected, and a double-barrel orifice was fashioned at the confluence of the right and left hepatic ducts in preparation for HJ. The remnant gallbladder and segments 4B and 5 of the liver were then resected, and a standard jejunojejunostomy and retrocolic HJ were performed with two Jackson-Pratt (JP) drains left in place. The patient recovered well postoperatively and was discharged on Day 5. Pathology of the samples obtained intraoperatively demonstrated small fragments of well-differentiated adenocarcinoma in the bile ducts extending to the en-face distal bile duct margin, with negative lymph nodes and benign liver with reactive changes, confirming malignancy extending only to the gallbladder and CBD, consistent with T2a staging. The patient had an uncomplicated recovery with a small hepatic abscess 3 weeks post-op, and JP drains were removed ~1.5 months post-op. The patient was then managed by medical oncology and started on an adjuvant 6-month course of capecitabine, on which she remains with no significant adverse effects to date.

## Discussion

Due to the significantly increased risk and incidence of gallbladder cancer with Mirizzi syndrome [9–11], and the anatomical distortions caused by inflammation secondary to Mirizzi syndrome, management of this condition is complicated. Mirizzi syndrome is thought to contribute to carcinogenesis by accelerating the inflammation–dysplasia–carcinoma sequence, specifically by causing biliary stasis, cholangitis, and inflammation that predisposes to cellular and molecular alterations that promote carcinogenesis [11]. Our patient likely had chronic cholestasis secondary to significant gallstone disease for an extended period, as there was significant inflammation and infection observed during her initial laparoscopic cholecystectomy. Diagnosis of the primary tumor site was also particularly challenging in this case, as distortions in the normal hepatobiliary anatomy secondary to Mirizzi syndrome prevented pathological confirmation of a gallbladder or bile duct primary site. Exceedingly rarely, cases of synchronous primary bile duct malignancy and primary gallbladder malignancy can arise [12], though this was deemed unlikely in our patient’s case, as there were no significant pathological differences in the tumor at different sampling sites and no other evidence of heterogeneity suggesting synchronous tumors. This case underscores the association between Mirizzi and biliary malignancy and illustrates the complex approach necessary to successfully resect tumors associated with Mirizzi syndrome.
